# Cerebrospinal fluid MMP-2 and TIMP-2 reflect blood–cerebrospinal fluid barrier dysfunction in Guillain–Barré syndrome and CIDP

**DOI:** 10.3389/fneur.2026.1993595

**Published:** 2026-10-28

**Authors:** Jiuheng Zhong, Jia Li, Ziqi Mo, Ya Zeng, Jiewei Peng, Peipei Zhu, Haiying Wang, Caiyun Mo, Yutao Liu, Yu Tu

**Affiliations:** 1 Zhuhai Campus of Zunyi Medical University, Zhuhai, Guangdong, China; 2 Zhuhai People's Hospital (The Affiliated Hospital of Beijing Institute of Technology), Zhuhai Clinical Medical College of Jinan University, Zhuhai, China; 3 The First School of Clinical Medicine, Guangdong Medical University, Zhanjiang, Guangdong, China

**Keywords:** blood–cerebrospinal fluid barrier, chronic inflammatory demyelinating polyneuropathy, Guillain–Barré syndrome, matrix metalloproteinases, tissue inhibitors of metalloproteinases

## Abstract

**Background:**

This study aimed to investigate patterns of matrix metalloproteinases (MMPs) and their tissue inhibitors (TIMPs) in Guillain–Barré syndrome (GBS) and chronic inflammatory demyelinating polyneuropathy (CIDP). It also examined how these patterns relate to dysfunction of the blood–cerebrospinal fluid barrier (BCB).

**Methods:**

This prospective observational study enrolled 86 participants: 33 patients with GBS, 34 with CIDP, and 19 with Alzheimer’s disease (AD) as controls. Enzyme-linked immunosorbent assay (ELISA) quantified MMP-2, MMP-9, TIMP-1, and TIMP-2 levels in blood and cerebrospinal fluid (CSF). Analyses included group comparisons, multivariable logistic regression, ROC curve analysis, and multivariable linear regression to assess classification value and association with CSF protein (CSF-Pro).

**Results:**

Compared to controls, patients with immune-mediated peripheral neuropathies had lower blood MMP-2 and TIMP-2 levels and higher blood MMP-9 levels. CSF MMP-2 and TIMP-2 levels were higher in CIDP than in GBS. Multivariable logistic regression showed CSF MMP-2 was associated with disease classification. ROC analysis revealed CSF MMP-2 moderately distinguished CIDP from GBS (AUC = 0.700). Multivariable linear regression showed CSF TIMP-2 was independently and negatively associated with CSF-Pro levels.

**Conclusion:**

The MMP-2/TIMP-2 axis may contribute to BCB dysfunction in immune-mediated peripheral neuropathies. CSF MMP-2 levels are associated with disease classification, while elevated CSF TIMP-2 levels may alleviate barrier damage. These biomarkers may serve as potential reference indicators for assessing barrier dysfunction and could provide a basis for differentiating GBS from CIDP.

## Introduction

Guillain–Barré syndrome (GBS) is an immune-mediated acute inflammatory peripheral neuropathy. It is characterized by electrophysiological changes, often involving demyelinating or axonal injury, blood–cerebrospinal fluid barrier (BCB) dysfunction, albumin cytologic dissociation in cerebrospinal fluid, and positive serum antiganglioside antibodies (1). Chronic Inflammatory Demyelinating Polyneuropathy (CIDP) is a chronic autoimmune disorder that affects the peripheral nervous system. GBS and CIDP share features, including immune-mediated myelin injury and inflammatory responses (2). They can be differentiated by mode of onset, electrophysiological examination, nerve ultrasound, cerebrospinal fluid findings, and immune markers (Peripheral nerve antibodies). However, some atypical cases show overlapping onset patterns, with electrophysiological findings falling between the two, making differentiation difficult (3).

Matrix metalloproteinases (MMPs) are calcium-dependent zinc endopeptidases; local inflammatory cells are the primary source of circulating MMPs. At lesion sites in GBS and CIDP, the recruitment and activation of inflammatory cells further promote MMP secretion (4). MMPs degrade basement membrane components and disrupt barrier function (5). TIMPs tightly regulate their activity; the TIMP subtypes differ in composition and function. TIMP-1 primarily targets and inhibits MMP-9, while TIMP-2 mainly regulates MMP-2; TIMP-3 can broadly inhibit multiple MMPs, and TIMP-4 is homologous to TIMP-2 and has similar functions (6). Imbalances between MMPs and TIMPs can disrupt the BCB, leading to demyelination and neuronal injury (7). GBS and CIDP share a similar background of immune-mediated peripheral nerve injury, yet they differ in disease course, treatment strategies, and prognosis. Clinical manifestations overlap in some early or atypical cases, so biomarkers are needed to support differential diagnosis. Previous studies have reported abnormal MMP and TIMP expression in both disorders, supporting a comparison of their expression profiles (8, 9). The MMP/TIMP system is also involved in other neuroinflammatory conditions (10). This study focuses on GBS and CIDP to assess the discriminative value of these biomarkers in a comparable disease background, rather than assuming that these alterations are disease-specific. Furthermore, this study confirms that elevation of CSF TIMP-1 levels in GBS may have prognostic value (11). Serum MMP-2 and MMP-9 levels have been reported to be increased in GBS (12). Altered plasma expression of MMP-2, MMP-9, TIMP-1, and TIMP-2 has been observed in CIDP (9). Therefore, this study focuses on TIMP-1 and TIMP-2. However, previous studies have mostly examined GBS or CIDP separately, and clinical studies comparing MMP/TIMP profiles in the serum and cerebrospinal fluid of patients with GBS and CIDP under the same experimental conditions and using standardized testing protocols remain limited.

This study aimed to explore the relationship between MMPs/TIMPs and BCB dysfunction in patients with GBS and CIDP, as well as in controls. By comparing levels of MMP-2, MMP-9, TIMP-1, and TIMP-2 in blood and CSF, we sought to evaluate the clinical value of MMPs as potential biomarkers of BCB dysfunction in GBS and CIDP.

MMP/TIMP profiles may provide complementary biological information, but further validation is needed to determine whether they offer additional discriminatory value beyond standard clinical and electrophysiological assessment.

## Materials and methods

### Research subjects

#### GBS and CIDP groups

Patients diagnosed with GBS or CIDP who presented to the Department of Neurology at Zhuhai People’s Hospital between January 2023 and November 2025 were consecutively enrolled. Inclusion criteria were: (1) age 18–80 years; (2) a confirmed clinical diagnosis of GBS or CIDP based on the Guillain–Barré and Miller-Fisher syndromes—new diagnostic classification (13) and the European Academy of Neurology/Peripheral Nerve Society guideline for CIDP (14); (3) no prior (13) treatment with corticosteroids, intravenous immunoglobulin, plasma exchange, or immunosuppressive agents; (4) no severe infection or other immune-related disease at onset; (5) provision of written informed consent.

Exclusion criteria were: (1) failure to complete lumbar puncture or obtain CSF samples; (2) presence of systemic infectious diseases, malignancies, severe cardiac, hepatic, or renal diseases, or hematologic disorders; (3) sequelae that interfered with assessment of disease severity in GBS or CIDP; (4) pregnancy or lactation; (5) refusal to provide informed consent.

#### Control group

Nineteen patients with Alzheimer’s disease (AD) hospitalized in the Department of Neurology at Zhuhai People’s Hospital between October 2023 and July 2025, all of whom underwent lumbar puncture for clinical evaluation, were included as disease controls. This group provided a reference for blood and CSF MMP/TIMP profiles in GBS and CIDP and facilitated exploration of alterations shared by the two diseases. We selected them primarily because CSF collection was ethically feasible; they were not healthy controls with normal barrier function.

The study followed the Declaration of Helsinki and received approval from the Ethics Committee of Zhuhai People’s Hospital (Approval No. (2023) Lun Shen [Yan] No. 53). All participants or legal representatives provided written informed consent. Clinical data and biological samples were used only for this study, and participant confidentiality was strictly protected. The study was registered in the Chinese Clinical Trial Registry (ChiCTR2400090403).

### Research methods

#### Baseline clinical characteristics

A detailed history was taken within 2 days after admission. Data collected included age, sex, height, weight, smoking and alcohol history, and comorbidities such as hypertension, diabetes mellitus, and cardiovascular or cerebrovascular disease.

#### Blood sample collection and routine laboratory testing

Fasting venous blood was sampled from the antecubital vein on the morning after admission, after a 10–12 h fast. Samples were placed into appropriate tubes—plain, EDTA anticoagulant, or heparinized—then centrifuged. Complete blood counts were measured by trained laboratory technicians using the Mindray 6800Plus analyzer. Biochemical indicators were assessed using an automatic chemistry analyzer (7,600 Automatic Analyzer). These included alanine aminotransferase (ALT), aspartate aminotransferase (AST), creatinine (CREA), blood urea nitrogen (BUN), triglycerides (TG), total cholesterol (TC), low-density lipoprotein cholesterol (LDL-C), high-density lipoprotein cholesterol (HDL-C), serum uric acid (S-UA), and homocysteine (HCY). All tests were performed on the same platform according to the manufacturer’s instructions.

#### Measurement of MMPs/TIMPs in serum and CSF

Two milliliters of CSF were obtained by lumbar puncture. Five milliliters of venous blood were collected simultaneously. We allowed blood samples to stand for 30 min, then centrifuged them at 4000 rpm for 5 min to separate the serum. We aliquoted all serum and CSF samples and stored them at −80 °C until analysis. Once all group samples were collected, serum and CSF concentrations of MMP-2, MMP-9, TIMP-1, and TIMP-2 were quantified using commercially available double-antibody sandwich ELISA kits (Wuhan EIAab). Dilution ratios were optimized in pilot experiments. Standard curves were generated using a series of standards. We measured absorbance at 450 nm for quantitative analysis. All samples were tested under identical conditions. The calibration curves for the samples are shown in Figure 1.

**Figure 1:**
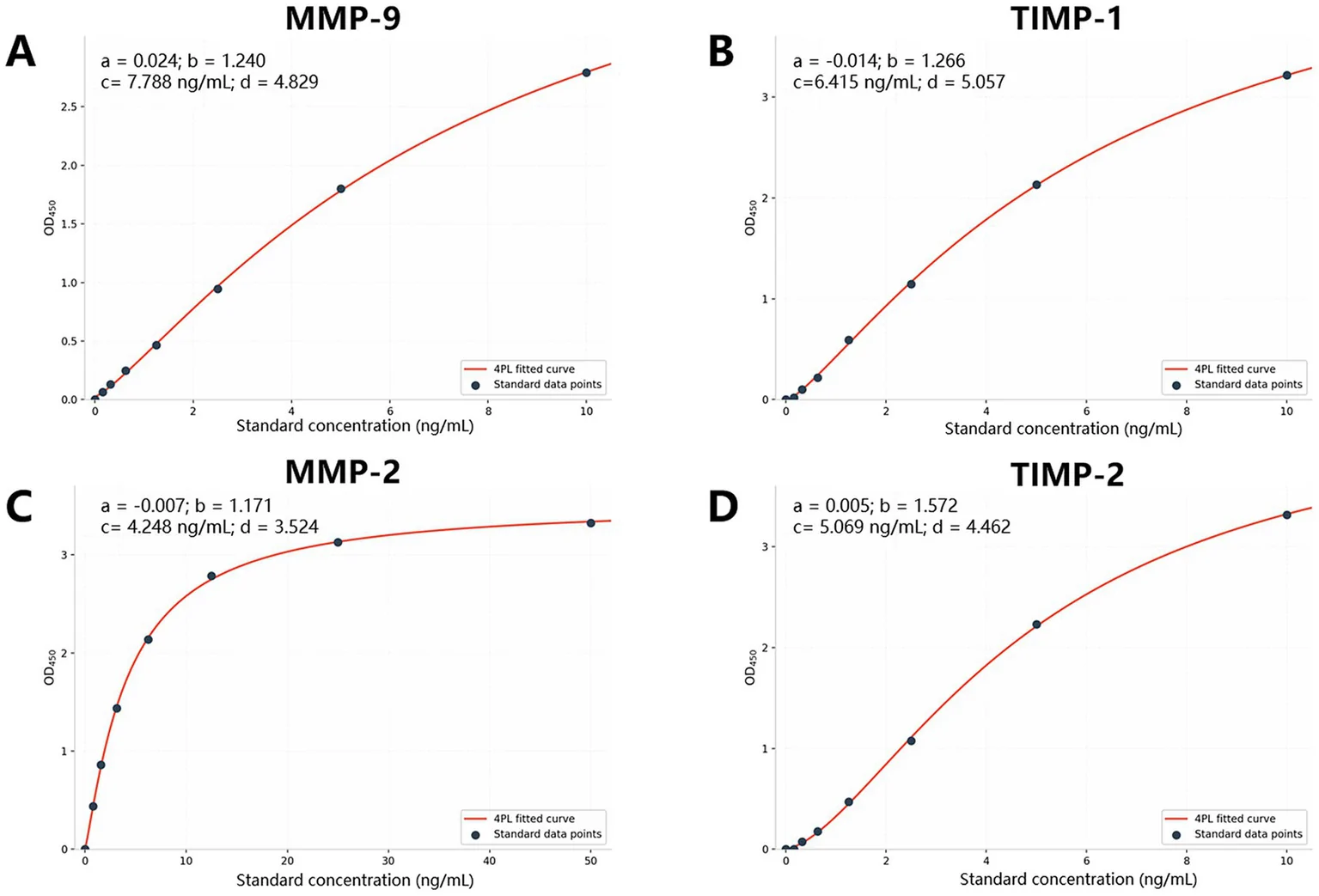
Four-parameter logistic (4PL) standard curves for ELISA quantification of MMP-9, TIMP-1, MMP-2, and TIMP-2. **(A)** MMP-9; **(B)** TIMP-1; **(C)** MMP-2; and **(D)** TIMP-2. Solid lines represent 4PL curves plotted using the parameters displayed in each panel, according to the equation *y* = *d* + (*a* - *d*) / [1 + (*x*/*c*) *b*]. Here, *x* denotes standard concentration (ng/mL), and *y* denotes optical density at 450 nm (OD450). Parameters *a* and *d* represent the lower and upper asymptotes, respectively; *b* is the slope parameter, and *c* is the concentration corresponding to the midpoint of the response range.

### Statistical methods

Statistical analyses were performed using SPSS version 27.0. Continuous variables were assessed for normality with the Shapiro–Wilk test. Most variables were not normally distributed, so results are shown as median (interquartile range) [Median (IQR)]. Group comparisons used the Kruskal–Wallis H test. Categorical variables are presented as counts and percentages, and compared using the χ^2^ test or Fisher’s exact test, as appropriate. One missing value in routine CSF parameters for the 80th patient was imputed using mean substitution. We considered the impact of this imputation limited, given the low missing rate. We used logistic regression to examine the association between biomarkers and disease classification (GBS vs. CIDP). Odds ratios (ORs) reflect the effect of a one-unit increase in each biomarker. We entered blood MMP-9 per 1,000 ng/mL because its range was broad. We used multivariable linear regression with CSF-Pro as the dependent variable to assess associations between biomarkers and CSF protein levels. ROC analysis evaluated diagnostic performance. AUC values, standard errors, and curve comparisons were calculated using the Hanley and McNeil method. All tests were two-tailed. We considered *p* < 0.05 statistically significant.

## Results

### Study participants and baseline characteristics

A total of 86 participants were enrolled: 67 patients with immune-mediated peripheral neuropathies—33 with GBS and 34 with CIDP—and 19 controls. The GBS group included 23 with acute inflammatory demyelinating polyneuropathy (AIDP), 6 with Miller–Fisher syndrome, and 4 with Bickerstaff brainstem encephalitis (BBE). The CIDP group included 21 with typical CIDP, 4 with multifocal acquired demyelinating sensory and motor neuropathy (MADSAM), 3 with distal acquired demyelinating symmetric neuropathy (DADS), and 6 with chronic immune sensory polyradiculopathy (CISP). Given the skewed distribution of disease duration, both the mean ± standard deviation and median are reported. Disease duration differed significantly between the groups: 11.61 ± 11.68 days (median, 8 days) in GBS and 1.53 ± 1.91 years (median, 1 year) in CIDP.

Baseline demographic and clinical characteristics are summarized in Table 1. No significant differences were observed between the GBS and CIDP groups in sex, age, comorbidities, lifestyle factors, body weight, or most laboratory variables (all *p* > 0.05), except that the GBS group had a higher white blood cell count than the CIDP group (*p* < 0.05). Compared with controls, patients in the disease groups had a higher proportion of males, were younger, had higher rates of smoking and alcohol consumption, greater body weight, and higher white blood cell counts, as well as lower HDL-C levels (all *p* < 0.05). No significant differences were found in other hepatic, renal, or metabolic laboratory parameters.

**Table 1:** Baseline characteristics of patients with GBS, CIDP, and controls.

Variable	GBS group (*n* = 33)	CIDP group (*n* = 34)	Control group (*n* = 19)	*P^1^* value	*P^2^* value
Male, *n* (%)	20 (60.61)	26 (76.47)	2 (10.53)	0.162	< 0.001^**^
Age, years	53.00 (38.00–66.50)	63.00 (57.50–70.00)	69.00 (62.00–78.00)	0.056	0.003^**^
Hypertension, *n* (%)	10 (30.30)	10 (29.41)	5 (26.31)	0.936	0.765
Diabetes mellitus, *n* (%)	6 (18.18)	2 (5.88)	1 (5.26)	0.121	0.401
History of stroke, *n* (%)	4 (12.12)	3 (8.82)	0 (0.00)	0.659	0.142
History of CAD, *n* (%)	3 (9.09)	1 (2.94)	1 (5.26)	0.288	0.907
Smoking, *n* (%)	12 (36.36)	15 (44.12)	0 (0.00)	0.550	< 0.001^***^
Alcohol abuse, *n* (%)	5 (15.15)	11 (32.35)	0 (0.00)	0.099	0.018^*^
Weight, kg	65.00 (55.50–75.50)	60.00 (50.75–69.25)	49.00 (46.00–60.00)	0.075	0.001^**^
WBC, ×10^9^/L	7.24 (5.60–9.32)	6.05 (4.89–7.68)	5.51 (4.77–6.27)	0.042^*^	0.023^*^
ALT, U/L	18.00 (12.00–32.50)	16.00 (12.75–26.25)	15.00 (12.00–17.00)	0.905	0.185
AST, U/L	18.00 (14.50–24.50)	18.00 (15.00–23.00)	19.00 (17.00–24.00)	0.782	0.275
CREA, μmol/L	66.00 (53.00–74.50)	66.00 (52.00–77.25)	66.00 (60.00–81.00)	0.851	0.625
BUN, mmol/L	5.70 (4.67–6.15)	5.25 (4.24–6.13)	5.30 (4.50–5.85)	0.231	0.864
S–UA, μmol/L	291.00 (254.50–407.50)	315.00 (273.75–370.75)	283.00 (252.00–344.00)	0.821	0.423
TG, mmol/L	1.34 (0.94–1.79)	1.08 (0.76–1.68)	1.10 (0.84–1.52)	0.419	0.450
CHOL, mmol/L	4.55 (3.82–5.40)	4.57 (3.93–5.65)	4.88 (4.06–5.48)	0.950	0.739
HDL, mmol/L	1.24 (0.96–1.49)	1.36 (1.07–1.64)	1.54 (1.18–1.81)	0.319	0.029^*^
LDL, mmol/L	2.62 (2.13–3.38)	2.50 (1.89–3.06)	2.73 (1.63–3.34)	0.422	0.942
HCY, μmol/L	12.00 (9.00–13.00)	12.00 (10.00–14.00)	10.00 (9.00–14.00)	0.289	0.224

Data are presented as median (P25–P75) for continuous variables and *n* (%) for categorical variables. *P*^1^ indicates comparison between GBS and CIDP groups; *P*^2^ indicates the comparison between the combined immune–mediated peripheral neuropathy group (GBS and CIDP) and the control group. ^*^p < 0.05, ^**^p < 0.01, ^***^p < 0.001. These *p* values are unadjusted. Global three–group comparisons with Bonferroni–adjusted post hoc tests are presented in Table 3.

### Comparison of routine cerebrospinal fluid parameters

Routine CSF parameters and blood biochemical indices among the GBS, CIDP, and control groups are shown in Table 2. Among patients with immune-mediated peripheral neuropathies, no significant differences were found between the GBS and CIDP groups in CSF cell differentials, CSF protein (CSF-Pro), CSF chloride, CSF glucose, or blood glucose levels (all *p* > 0.05). In contrast, blood chloride levels were significantly higher in the CIDP group than in the GBS group (*p* = 0.005). Compared with controls, patients showed higher proportions of CSF mononuclear cells and CSF protein levels, whereas CSF chloride and glucose levels were lower (all *p* < 0.05). Blood glucose levels did not differ significantly between groups.

**Table 2:** Comparison of routine CSF parameters and levels of MMPs and TIMPs in blood and CSF among patients with GBS, CIDP, and controls, Median (IQR).

Variable	GBS group (*n* = 33)	CIDP group (*n* = 34)	Control group (*n* = 19)	*P^1^* value	*P^2^* value
Mononuclear cells, %	69.00 (0.00–85.10)	50.00 (0.00–75.00)	0.00 (0.00–50.00)	0.167	0.008^**^
Polymorphonuclear cells, %	14.80 (0.00–30.50)	5.00 (0.00–26.00)	0.00 (0.00–14.88)	0.523	0.058
CSF–Pro, mg/dL	637.5 (338.05–997.20)	456.55 (332.75–757.15)	367.00 (290.00–415.00)	0.139	0.002^**^
CSF–CL, mmol/L	124.00 (122.50–126.50)	125.00 (123.75–126.00)	126.00 (124.00–127.00)	0.356	0.033^*^
CSF–GLU, mmol/L	3.70 (3.30–4.60)	3.60 (3.14–4.05)	3.40 (3.00–3.70)	0.337	0.029^*^
GLU–Blood, mmol/L	5.30 (4.35–7.10)	5.23 (4.58–6.34)	5.38 (4.54–6.90)	0.990	0.925
CL–Blood, mmol/L	104.00 (102.00–107.00)	107.00 (105.00–109.00)	107.00 (105.00–109.00)	0.005^**^	0.049
MMP–2 (Blood), ng/mL	14.01 (11.07–16.35)	13.51 (11.63–16.89)	19.71 (15.30–21.59)	0.945	< 0.001^***^
MMP–2 (CSF), ng/mL	2.74 (2.14–2.99)	3.06 (2.65–3.63)	2.60 (1.55–3.61)	0.005^**^	0.252
MMP–9 (Blood), ng/mL	4077.63 (2255.78–12371.71)	3560.95 (1732.71–6331.71)	1660.93 (1528.60–1828.01)	0.073	< 0.001^***^
MMP–9 (CSF), ng/mL	1.11 (0.61–2.00)	1.18 (0.67–6.61)	1.04 (0.48–5.81)	0.328	0.815
TIMP–1 (Blood), ng/mL	61.35 (39.60–92.09)	41.17 (30.09–54.18)	37.55 (28.38–78.73)	0.021^*^	0.578
TIMP–1 (CSF), ng/mL	9.11 (4.33–17.39)	14.31 (8.90–22.28)	10.53 (3.79–16.51)	0.042^*^	0.221
TIMP–2 (Blood), ng/mL	78.75 (62.93–110.25)	82.15 (61.30–112.72)	124.26 (78.47–152.39)	0.930	0.005^**^
TIMP–2 (CSF), ng/mL	42.00 (31.22–58.62)	63.86 (44.09–71.13)	23.11 (11.91–40.05)	0.014^*^	< 0.001^***^
MMP–2 (Blood/CSF ratio)	5.28 (4.38–6.67)	4.31 (3.64–5.60)	7.29 (5.36–12.91)	0.052	< 0.001^***^
TIMP–2 (Blood/CSF ratio)	1.72 (1.27–3.32)	1.40 (1.11–1.96)	3.83 (2.64–10.44)	0.069	< 0.001^***^
MMP–9 (Blood/CSF ratio)	4209.16 (1178.61–14134.80)	2229.70 (627.05–5852.54)	1459.64 (247.02–3375.70)	0.077	0.108
TIMP–1 (Blood/CSF ratio)	6.90 (2.92–9.10)	2.46 (1.65–4.52)	4.58 (2.98–10.05)	0.002^**^	0.305
MMP–2/TIMP–2 (CSF)	0.06 (0.05–0.08)	0.05 (0.04–0.07)	0.09 (0.08–0.18)	0.316	< 0.001^***^
MMP–2/TIMP–2 (Blood)	0.16 (0.14–0.20)	0.16 (0.14–0.20)	0.17 (0.14–0.19)	0.735	0.476
MMP–9/ TIMP–1 (Blood)	95.33 (55.41–141.64)	77.65 (37.39–123.16)	40.54 (21.98–40.54)	0.281	< 0.001^***^
MMP–9/ TIMP–1 (CSF)	0.09 (0.06–0.27)	0.08 (0.05–0.30)	0.23 (0.04–0.83)	0.841	0.390

Data are presented as median (P25–P75) for continuous variables and *n* (%) for categorical variables. *P*^1^ indicates comparison between GBS and CIDP groups; P2 indicates the comparison between the combined immune-mediated peripheral neuropathy group (GBS and CIDP) and the control group. ^*^p < 0.05, ^**^p < 0.01, ^***^p < 0.001. These *p* values are unadjusted. Global three-group comparisons with Bonferroni-adjusted post hoc tests are presented in Table 3.

### Comparison of plasma and cerebrospinal fluid MMPs/TIMPs levels among the GBS, CIDP, and control groups

For individual biomarkers (Table 2), partial differences were observed between the GBS and CIDP groups. Blood TIMP-1 levels were higher in the GBS group than in the CIDP group (*p* = 0.021), whereas CSF MMP-2 (*p* = 0.005), TIMP-1 (*p* = 0.042), and TIMP-2 (*p* = 0.014) levels were lower in the GBS group than in the CIDP group. No significant differences were observed for the remaining biomarkers (all *p* > 0.05).

Three-group comparisons (Table 3) showed significant differences in blood MMP-2 (*p* < 0.001) and CSF MMP-2 (*p* = 0.018). Bonferroni-adjusted pairwise comparisons showed that both the GBS and CIDP groups had lower blood and CSF MMP-2 levels than controls. Blood MMP-9 differed significantly among the three groups overall (*p* < 0.001), but no pairwise comparison remained significant after correction, whereas CSF MMP-9 showed no significant intergroup difference (*p* = 0.628). Among the inhibitors, blood TIMP-2 (*p* = 0.021) and CSF TIMP-2 (*p* < 0.001) differed significantly among groups; both the GBS and CIDP groups had lower blood TIMP-2 levels than controls, whereas CSF TIMP-2 was higher in CIDP than in controls. Neither blood nor CSF TIMP-1 differed significantly among groups (*p* = 0.058 and *p* = 0.057, respectively).

**Table 3:** Comparison of MMPs and TIMPs levels among GBS, CIDP, and control groups (Kruskal–Wallis test with Bonferroni correction), median (IQR).

Variable	GBS (*n* = 33)	CIDP (*n* = 34)	Control (*n* = 19)	Overall *p* value†	Significant pairwise comparisons‡
MMP-2 (Blood), ng/mL	14.01 (11.07–16.35)	13.51 (11.63–16.89)	19.71 (15.30–21.59)	< 0.001	GBS vs. Control; CIDP vs. Control
MMP-2 (CSF), ng/mL	2.74 (2.14–2.99)	3.06 (2.65–3.63)	2.60 (1.55–3.61)	0.018	GBS vs. Control; CIDP vs. Control
MMP-9 (Blood), ng/mL	4077.63 (2255.78–12371.71)	3560.95 (1732.71–6331.71)	1660.93 (1528.60–1828.01)	< 0.001	No pairwise comparisons remained significant after Bonferroni correction.
MMP-9 (CSF), ng/mL	1.11 (0.61–2.00)	1.18 (0.67–6.61)	1.04 (0.48–5.81)	0.628	–
TIMP-1 (Blood), ng/mL	61.35 (39.60–92.09)	41.17 (30.09–54.18)	37.55 (28.38–78.73)	0.058	–
TIMP-1 (CSF), ng/mL	9.11 (4.33–17.39)	14.31 (8.90–22.28)	10.53 (3.79–16.51)	0.057	–
TIMP-2 (Blood), ng/mL	78.75 (62.93–110.25)	82.15 (61.30–112.72)	124.26 (78.47–152.39)	0.021	GBS vs. Control; CIDP vs. Control
TIMP-2 (CSF), ng/mL	42.00 (31.22–58.62)	63.86 (44.09–71.13)	23.11 (11.91–40.05)	< 0.001	CIDP vs. Control
MMP-2 (Blood/CSF ratio)	5.28 (4.38–6.67)	4.31 (3.64–5.60)	7.29 (5.36–12.91)	< 0.001	CIDP vs. Control
MMP-9 (Blood/CSF ratio)	4209.16 (1178.61–14134.80)	2229.70 (627.05–5852.54)	1459.64 (247.02–3375.70)	0.066	-
TIMP-2 (Blood/CSF ratio)	1.72 (1.27–3.32)	1.40 (1.11–1.96)	3.83 (2.64–10.44)	< 0.001	GBS vs. Control; CIDP vs. Control
TIMP-1 (Blood/CSF ratio)	6.90 (2.92–9.10)	2.46 (1.65–4.52)	4.58 (2.98–10.05)	0.006	GBS vs. CIDP
MMP-2/TIMP-2 (Blood)	0.16 (0.14–0.20)	0.16 (0.14–0.20)	0.17 (0.14–0.19)	0.727	–
MMP-2/TIMP-2 (CSF)	0.06 (0.05–0.08)	0.05 (0.04–0.07)	0.09 (0.08–0.18)	< 0.001	GBS vs. Control; CIDP vs. Control
MMP-9/TIMP-1 (Blood)	95.33 (55.41–141.64)	77.65 (37.39–123.16)	40.54 (21.98–40.54)	0.001	GBS vs. Control; CIDP vs. Control
MMP-9/TIMP-1 (CSF)	0.09 (0.06–0.27)	0.08 (0.05–0.30)	0.23 (0.04–0.83)	0.678	–

^†^Overall *p* values were calculated using the Kruskal–Wallis test.

^‡^Pairwise comparisons were performed using Bonferroni correction for multiple testing.

The adjusted significance level was set at *p* < 0.017 (0.05/3).

Only statistically significant comparisons after Bonferroni correction are shown.

For ratio-based indicators (Tables 2, 3), the GBS group had a higher TIMP-1 blood/CSF ratio than the CIDP group (*p* = 0.002). Three-group comparisons showed significant differences in the MMP-2 blood/CSF ratio (*p* < 0.001), the TIMP-2 blood/CSF ratio (*p* < 0.001), and the CSF MMP-2/TIMP-2 ratio (*p* < 0.001), with lower values in the GBS and CIDP groups than in controls. In addition, the blood MMP-9/TIMP-1 ratio also differed significantly among groups (*p* = 0.001), with higher values in both the GBS and CIDP groups than in controls. The remaining ratio-based indicators, including the MMP-9 blood/CSF ratio, blood MMP-2/TIMP-2 ratio, and CSF MMP-9/TIMP-1 ratio, were not significantly different among groups (all *p* > 0.05).

### Multivariable logistic regression analysis of MMPs/TIMPs in the GBS and CIDP groups

To further evaluate the associations between MMPs/TIMPs and disease classification, GBS vs. CIDP (with CIDP as the reference category) was used as the dependent variable, and blood and CSF levels of MMP-2, MMP-9, TIMP-1, and TIMP-2 were entered simultaneously into a multivariable logistic regression model. After adjustment for the other biomarkers, CSF MMP-2 was associated with disease classification (OR = 5.806, 95% CI 1.258–26.790, *p* = 0.024). In addition, blood TIMP-1 was also associated with disease classification (OR = 0.964, 95% CI 0.933–0.996, *p* = 0.026), suggesting that higher blood TIMP-1 levels were associated with GBS rather than CIDP. In contrast, blood MMP-2, blood MMP-9, CSF MMP-9, CSF TIMP-1, and both blood and CSF TIMP-2 were not significantly associated with disease classification (all *p* > 0.05). Because blood MMP-9 values spanned a broad numerical range, this biomarker was entered into the model as a 1,000 ng/mL increase (Table 4).

**Table 4:** Multivariable logistic regression analysis for disease classification (GBS vs. CIDP).

Variable	OR	95% CI	*p* value
MMP-2 (Blood) (ng/mL)	0.849	0.612–1.178	0.327
MMP-2 (CSF) (ng/mL)	5.806	1.258–26.790	0.024^*^
MMP-9 (Blood), per 1,000 ng/mL	0.757	0.375–1.527	0.437
MMP-9 (CSF) (ng/mL)	0.996	0.891–1.113	0.948
TIMP-1 (Blood) (ng/mL)	0.964	0.933–0.996	0.026^*^
TIMP-1 (CSF) (ng/mL)	1.012	0.942–1.087	0.746
TIMP-2 (Blood) (ng/mL)	1.047	0.997–1.100	0.066
TIMP-2 (CSF) (ng/mL)	0.979	0.945–1.014	0.236

^*^p < 0.05. CIDP was used as the reference category. Continuous variables were entered into the model as continuous variables. Due to the large magnitude of blood MMP-9 concentrations, this variable was rescaled and entered as a per-1000-ng/mL increase.

### ROC curve analysis of MMPs/TIMPs for differentiating GBS and CIDP

Among blood biomarkers (Figure 2A; Table 5A), TIMP-1 (AUC = 0.664, 95% CI 0.538–0.775, *p* = 0.017) and MMP-9 (AUC = 0.627, 95% CI 0.501–0.743, *p* = 0.067) showed some ability to differentiate GBS from CIDP, whereas MMP-2 (AUC = 0.505, 95% CI 0.380–0.629) and TIMP-2 (AUC = 0.506, 95% CI 0.381–0.631) showed discriminatory performance close to chance.

**Figure 2:**
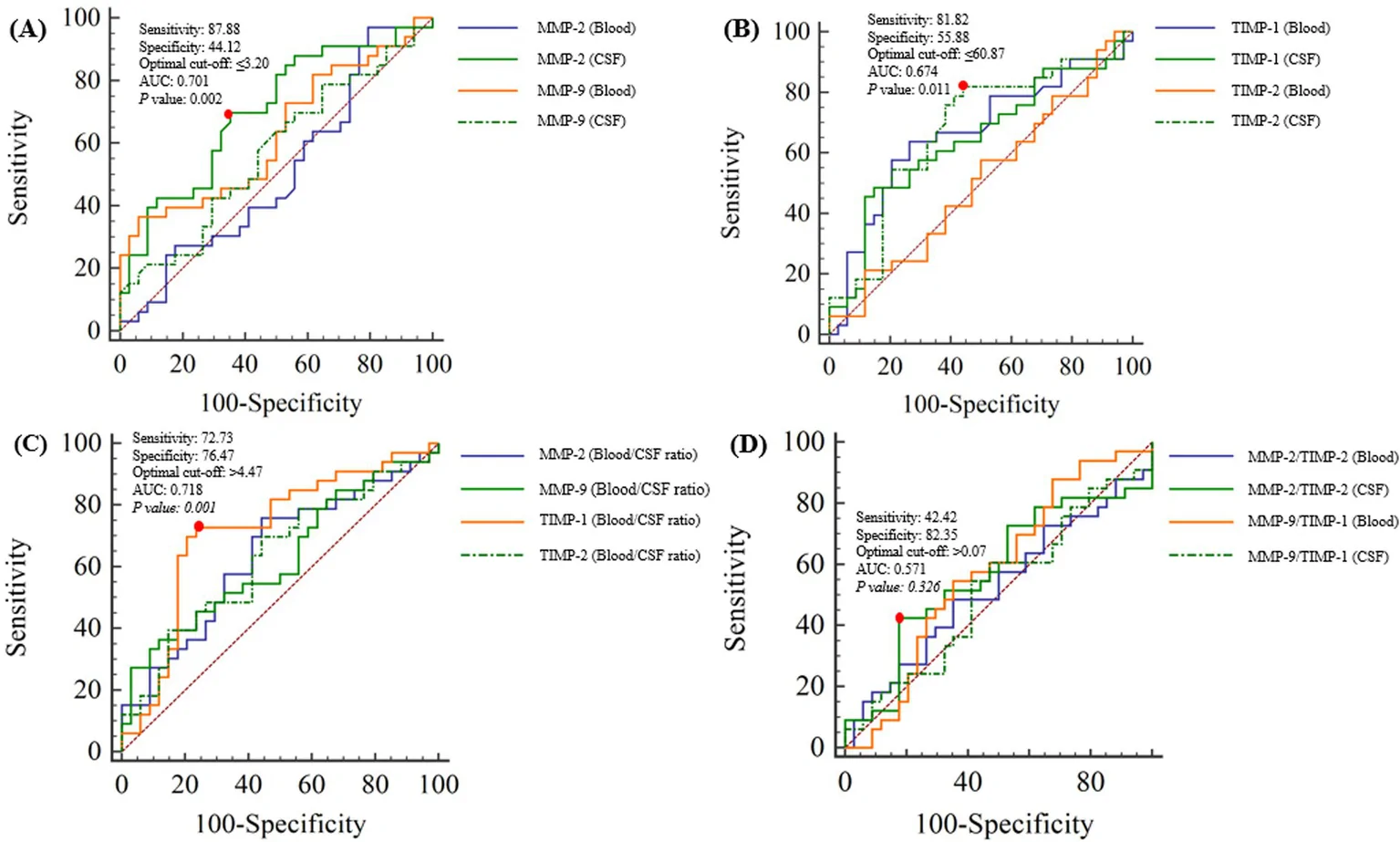
Diagnostic performance of blood and CSF MMPs/TIMPs in distinguishing GBS from CIDP. **(A)** ROC curves of MMP-2 and MMP-9 levels in blood and CSF. **(B)** ROC curves of TIMP-1 and TIMP-2 levels in blood and CSF. **(C)** ROC curves of blood/CSF ratios of MMP-2, MMP-9, TIMP-1, and TIMP-2. **(D)** ROC curves of MMP/TIMP ratios (MMP-2/TIMP-2 and MMP-9/TIMP-1) in blood and CSF. The CIDP group served as the reference category, and the GBS group was defined as the positive classification for ROC analysis. Red dots indicate optimal cut-off values based on the maximum Youden index. Sensitivity, specificity, AUC, and *p* values are presented in each panel.

**Table 5:** ROC analysis of MMPs and TIMPs for differentiating CIDP from GBS.

A. ROC analysis of individual MMPs/TIMPs and their ratios						
MMPs/TIMPs and their ratios	AUC (95% CI)	Youden index	Optimal cut-off	Sensitivity (%)	Specificity (%)	*P value*
MMP-2 (Blood)	0.505 (0.380–0.629)	0.176	17.93	96.97	20.59	0.946
MMP-2 (CSF)	0.700 (0.576–0.806)	0.344	2.90	69.70	64.71	0.002^**^
MMP-9 (Blood)	0.627 (0.501–0.743)	0.305	7787.24	36.36	94.12	0.067
MMP-9 (CSF)	0.570 (0.443–0.690)	0.141	2.08	78.79	35.29	0.328
TIMP-1 (Blood)	0.664 (0.538–0.775)	0.372	51.04	73.53	51.04	0.017^*^
TIMP-1 (CSF)	0.644 (0.518–0.758)	0.338	7.61	48.48	85.29	0.037^*^
TIMP-2 (Blood)	0.506 (0.381–0.631)	0.094	57.77	21.21	88.24	0.931
TIMP-2 (CSF)	0.674 (0.549–0.784)	0.377	60.87	81.82	55.88	0.011^*^
MMP-2 (Blood/CSF ratio)	0.638 (0.512–0.752)	0.316	4.34	75.76	55.88	0.045^*^
MMP-9 (Blood/CSF ratio)	0.626 (0.499–0.741)	0.246	9402.95	36.36	88.24	0.070
TIMP-2 (Blood/CSF ratio)	0.629 (0.503–0.744)	0.256	1.47	69.70	55.88	0.062
TIMP-1 (Blood/CSF ratio)	0.718 (0.595–0.821)	0.492	4.47	72.73	76.47	*<*0.001^***^
MMP-2/TIMP-2 (Blood)	0.524 (0.398–0.648)	0.132	0.15	48.48	64.71	0.734
MMP-2/TIMP-2 (CSF)	0.571 (0.444–0.692)	0.248	0.07	42.42	82.35	0.326
MMP-9/TIMP-1 (Blood)	0.577 (0.450–0.697)	0.202	42.45	87.88	32.35	0.284
MMP-9/TIMP-1 (CSF)	0.514 (0.389–0.638)	0.136	0.08	60.61	52.94	0.843
^*^p < 0.05, ^**^p < 0.01, ^***^p < 0.001. Optimal cut-off values were determined using the Youden index. Sensitivity and specificity were calculated at the optimal cut-off point.						

B. Pairwise comparison of ROC curves (Hanley & McNeil method)				
Comparison	ΔAUC	95% CI	Z	*p* value
MMP-2 (Blood) vs. MMP-2 (CSF)	0.195	0.017–0.373	2.148	**0.032**
TIMP-1 (Blood) vs. TIMP-2 (Blood)	0.158	0.0318–0.284	2.455	**0.014**
TIMP-1 (Blood) vs. TIMP-1 (CSF)	0.020	−0.148–0.187	0.230	0.818
TIMP-2 (Blood) vs. TIMP-2 (CSF)	0.168	−0.007–0.343	1.878	0.060
All other pairwise comparisons	-	-	-	>0.05

AUCs and standard errors were calculated using the Hanley & McNeil method.

Differences between ROC curves were compared using the Hanley & McNeil approach.

Bold values indicate statistically significant differences (*p* < 0.05).

Among CSF biomarkers (Figures 2A,B; Table 5A), MMP-2 showed the highest diagnostic performance (AUC = 0.700, 95% CI 0.576–0.806, *p* = 0.002), followed by TIMP-2 (AUC = 0.674, *p* = 0.011) and TIMP-1 (AUC = 0.644, *p* = 0.037), whereas MMP-9 showed relatively poor discrimination (AUC = 0.570, *p* = 0.328).

When the ROC curves of the same biomarkers in blood and CSF were compared (Table 5B), the AUC for CSF MMP-2 was significantly higher than that for blood MMP-2 (ΔAUC = 0.195, 95% CI 0.017–0.373, *p* = 0.032). No significant differences were found between blood and CSF for MMP-9, TIMP-1, or TIMP-2 (all *p* > 0.05).

In addition, analysis of blood/CSF ratio-based indicators (Figure 2C; Table 5A) showed that the TIMP-1 blood/CSF ratio had relatively favorable diagnostic performance (AUC = 0.718, 95% CI 0.595–0.821, *p* < 0.001). For MMP/TIMP ratio-based indicators (Figure 2D; Table 5A), including MMP-2/TIMP-2 and MMP-9/TIMP-1, diagnostic performance was generally low, with AUCs close to 0.5 and no meaningful discriminatory ability.

### Association between MMPs/TIMPs and CSF protein levels

To further assess the relationships among blood and CSF MMPs/TIMPs and CSF protein levels, multivariable linear regression was performed with CSF-Pro as the dependent variable, and blood and CSF levels of MMP-2, MMP-9, TIMP-1, and TIMP-2 were entered simultaneously into the model (Figure 3). CSF TIMP-2 was significantly and negatively associated with CSF-Pro (β = −0.286, 95% CI − 0.542 to − 0.030, *p* = 0.029). In contrast, blood and CSF MMP-2, MMP-9, and TIMP-1, as well as blood TIMP-2, were not significantly associated with CSF-Pro (all *p* > 0.05). In addition, CSF MMP-2 showed a borderline positive association with CSF-Pro (β = 0.338, *p* = 0.064), but this did not reach statistical significance. Variance inflation factor (VIF) values for all variables were < 5, indicating no significant multicollinearity in the model.

**Figure 3:**
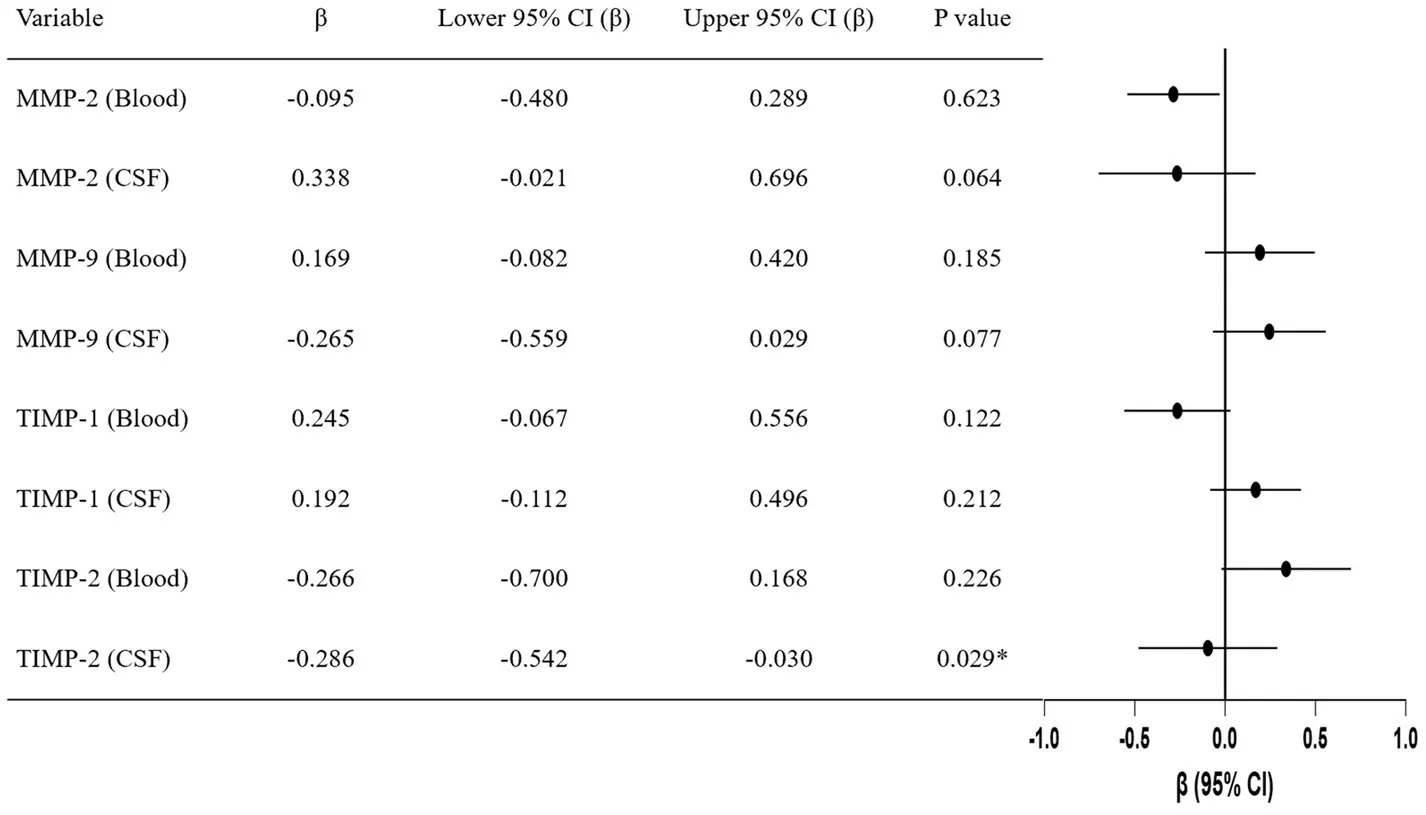
Multivariable linear regression analysis of biomarkers associated with CSF-Pro. Standardized regression coefficients (*β*) and 95% confidence intervals are shown. All biomarkers were simultaneously entered into the multivariable linear regression model. The vertical line represents *β* = 0. Multicollinearity was assessed using the variance inflation factors (VIFs), and all VIFs were < 5. *p < 0.05.

## Discussion

This study systematically evaluated MMP and TIMP expression in blood and CSF from patients with immune-mediated peripheral neuropathies. Although GBS and CIDP overlapped in many clinical variables, the MMP/TIMP system—particularly in CSF—showed distinct expression patterns. Multivariable logistic regression showed that, after adjusting for other MMP/TIMP markers, CSF MMP-2 remained associated with disease classification. In contrast, multivariable linear regression indicated that CSF TIMP-2 was negatively associated with CSF-Pro levels. These findings suggest that the MMP-2/TIMP-2 axis may be involved in BCB dysfunction in immune-mediated peripheral neuropathies and may serve as a potential biomarker.

### MMPs/TIMPs imbalance and BCB dysfunction

The integrity of the BCB depends on the stability of tight junctions in the choroid plexus epithelium and the basement membrane. Disruption of this structure may increase barrier permeability and elevate CSF protein levels (15). Elevated CSF protein is considered an important marker of increased BCB permeability and may reflect passive leakage of plasma proteins into the CSF (16). MMPs are important extracellular matrix-degrading enzymes that can contribute to barrier disruption by degrading tight junction proteins and basement membrane components, whereas their activity is tightly regulated by TIMPs (17). Previous studies have shown that an imbalance between MMPs and TIMPs is closely associated with inflammatory responses and barrier injury in a variety of neurological disorders (18). In the present study, altered expression patterns of MMPs/TIMPs were observed in both blood and CSF in GBS and CIDP, suggesting that protease–inhibitor imbalance may contribute to BCB dysfunction in immune-mediated peripheral neuropathies.

### Inflammatory activation of the MMP-9/TIMP-1 axis

With respect to the MMP-9/TIMP-1 axis, our results suggest that alterations in this axis primarily reflect inflammatory activation. MMP-9 is an important inflammation-related metalloproteinase that can increase barrier permeability by degrading tight junction proteins such as occludin and claudin, as well as extracellular matrix components (19). In this study, blood MMP-9 differed significantly among the three groups overall, but no significant pairwise differences remained after Bonferroni correction; moreover, CSF MMP-9 did not differ significantly among the three groups. These findings suggest that MMP-9 alterations mainly reflect inflammatory activation rather than disease-specific changes associated with disease classification. Previous studies have also reported elevated MMP-9 levels in GBS and CIDP (9, 20). However, because MMP-9 can be increased in various neuroinflammatory conditions, its diagnostic specificity is relatively limited (21). As its major endogenous inhibitor, TIMP-1 may regulate inflammation-related proteolytic activity (22). In the present study, TIMP-1 did not remain significantly different after multiple-comparison correction, consistent with previous reports, suggesting that the MMP-9/TIMP-1 axis may primarily regulate inflammation but has limited value in differentiating GBS from CIDP (11, 23). Further analysis showed that the blood MMP-9/TIMP-1 ratio tended to be elevated in both GBS and CIDP, suggesting enhanced proteolytic activity in the peripheral inflammatory milieu, which may promote extracellular matrix degradation and inflammation-associated barrier injury (7, 24).

### Role of the MMP-2/TIMP-2 axis in BCB remodeling

Our findings further suggest that the MMP-2/TIMP-2 axis may better reflect BCB dysfunction associated with GBS and CIDP. Although MMP-2 and MMP-9 both belong to the gelatinase family, they differ in their biological properties. MMP-2 is typically expressed as a relatively stable proenzyme and maintains a relatively stable basal level in CSF (25). Its activity is specifically regulated by TIMP-2, which both activates pro-MMP-2 and restrains excessive proteolysis (22). By contrast, MMP-9 rises rapidly during acute inflammation and is readily cleared by protease-inhibitory systems, such as α2-macroglobulin (26). Previous studies have also shown that MMP-2 contributes to barrier remodeling by degrading tight junction proteins such as Claudin-5 and Occludin (27).

In this study, compared with controls, patients with GBS and CIDP had lower blood MMP-2 levels, accompanied by decreased TIMP-2 levels, suggesting an altered systemic protease–inhibitor balance. In CSF, both MMP-2 and TIMP-2 were higher in CIDP than in GBS, and CSF MMP-2 was associated with disease classification in multivariable logistic regression. From a disease-course perspective, GBS is typically an acute monophasic disorder. In contrast, CIDP is characterized by chronic or relapsing immune-mediated inflammation accompanied by ongoing injury and repair of nerve roots and proximal nerves. In this chronic inflammatory microenvironment, MMP-2 may participate in basement membrane remodeling and barrier regulation, accompanied by compensatory changes in TIMP-2.

In addition, CSF TIMP-2 was negatively associated with CSF-Pro, suggesting that TIMP-2 may, at least in part, regulate BCB permeability. Experimental studies support this possibility. For example, Tang et al. reported that TIMP-2 reduced barrier leakage and improved neurological outcomes in a traumatic brain injury model (28). At the same time, Xu et al. showed that TIMP-2 maintained barrier integrity in an intracerebral hemorrhage model by regulating MMP-2 activity (29). These findings suggest that TIMP-2 may exert a protective regulatory role in maintaining barrier stability.

Moreover, we observed a decreased CSF MMP-2/TIMP-2 ratio, suggesting compensatory enhancement of inhibitory regulation in the local microenvironment, which may limit excessive extracellular matrix (ECM) degradation and barrier injury (30, 31). This finding further suggests that the protease–inhibitor system may exhibit layered regulation between peripheral inflammatory activation and local barrier control at the nerve root level.

### Diagnostic implications of CSF biomarkers

We further compared the discriminative performance of MMPs/TIMPs in blood and CSF. CSF MMP-2 showed moderate discrimination between CIDP and GBS, with a numerically higher AUC than blood MMP-2. This suggests that, compared with blood biomarkers, cerebrospinal fluid biomarkers may better reflect local pathological changes in the microenvironment of the nerve roots and proximal nerves. Previous studies have reported that in HIV-1-associated neurocognitive disorders, MMP-2 and MMP-9 activities are significantly elevated in CSF but not in peripheral blood (32); in multiple sclerosis, CSF MMP-9 has similarly been associated with inflammatory activity (33); and in varicella-zoster virus-related central nervous system infection, CSF MMPs are also markedly increased (34). Together, these findings indicate that alterations in CSF MMPs/TIMPs may more directly reflect local pathological processes within the nervous system. In summary, based on the existing literature and the results of this study, we speculate that changes in cerebrospinal fluid MMPs/TIMPs may reflect localized pathological processes within the nervous system. Within the proteolytic regulatory system, the MMP/TIMP system is a core pathway involved in extracellular matrix degradation and barrier damage. Proteolytic regulation in neuroinflammation is jointly mediated by multiple protease systems, rather than by a single pathway acting independently. In addition to the MMP family, other proteases, such as extracellular granzymes, also participate in extracellular matrix remodeling, activation of inflammatory signaling pathways, and regulation of BCB integrity (35).

### Limitations

This study has several limitations. First, the single-center design, small sample size, and lack of an independent validation cohort limit statistical power, model stability, and generalizability; therefore, the findings should be considered exploratory. Second, the cross-sectional design, without longitudinal follow-up or repeated CSF sampling, precludes assessment of temporal changes in these biomarkers. The absence of data on the 24-h intrathecal IgG synthesis rate and albumin quotient (QAlb) also limits assessment of intrathecal immune activity and blood–CSF barrier function. Moreover, the sources underlying the observed changes in MMP-2 and TIMP-2 concentrations remain uncertain. Third, differences in age and sex distributions between the AD control group and the GBS/CIDP groups may introduce confounding, while including only two male controls limits the reliability of sex-stratified analyses. Larger prospective longitudinal studies with better-matched groups, incorporating measures of barrier function and functional assays, are needed to clarify the biological significance of these biomarkers.

## Conclusion

In conclusion, we observed distinct MMP/TIMP expression patterns in patients with GBS and CIDP. CSF MMP-2 was associated with disease classification, whereas CSF TIMP-2 was negatively correlated with cerebrospinal fluid protein levels. Elevated CSF TIMP-2 levels may alleviate barrier damage. These findings suggest that the MMP-2/TIMP-2 axis may be involved in BCB dysfunction in immune-mediated peripheral neuropathies and may serve as a potential biomarker for disease differentiation and barrier function assessment.

## Data Availability

The original contributions presented in the study are included in the article/supplementary material, further inquiries can be directed to the corresponding authors.
